# Predicting pregnancy loss and its determinants among reproductive-aged women using supervised machine learning algorithms in Sub-Saharan Africa

**DOI:** 10.3389/fgwh.2025.1456238

**Published:** 2025-02-10

**Authors:** Tirualem Zeleke Yehuala, Sara Beyene Mengesha, Nebebe Demis Baykemagn

**Affiliations:** Department Health Informatics, Institute of Public Health, College of Medicine and Health Sciences, University of Gondar, Gondar, Ethiopia

**Keywords:** prediction, reproductive-aged women, pregnancy loss, machine learning, Sub-Saharan Africa

## Abstract

**Background:**

Pregnancy loss is a significant public health issue globally, particularly in Sub-Saharan Africa (SSA), where maternal health outcomes continue to be a major concern. Despite notable progress in improving maternal health, pregnancy-related complications, including s due to miscarriages, stillbirths, and induced abortions, continue to impact women's health, social wellbeing, and economic stability in the region. This study aims to identify the key predictors of pregnancy loss and develop effective predictive models for pregnancy loss among reproductive-aged women in SSA.

**Methods:**

We derived the data for this cross-sectional study from the most recent Demographic and Health Survey of Sub-Saharan African countries. Python software was used to process the data, and machine learning techniques such as Random Forest, Decision Tree, Logistic Regression, Extreme Gradient Boosting, and Gaussian were applied. The performance of the predictive models was evaluated using several standard metrics, including the ROC curve, accuracy score, precision, recall, and F-measure.

**Result:**

The final experimental results indicated that the Random Forest model performed the best in predicting pregnancy loss, achieving an accuracy of 98%, precision of 98%, F-measure of 83%, ROC curve of 94%, and recall of 77%. The Gaussian model had the lowest classification accuracy, with an accuracy of 92.64% compared to the others. Based on SHPY values, unmarried women may be more likely to experience pregnancy loss, particularly in contexts where premarital pregnancies are stigmatized. The use of antenatal care and family planning services can significantly impact the risk of pregnancy loss. Women from lower-income backgrounds may face challenges in accessing prenatal care or safe reproductive health services, leading to higher risks of loss. Additionally, higher levels of education are often correlated with increased awareness of family planning methods and better access to healthcare, which can reduce the likelihood of unintended pregnancy loss.

**Conclusion:**

The Random Forest machine learning model demonstrates greater predictive power in estimating pregnancy loss risk factors. Machine learning can help facilitate early prediction and intervention for women at high risk of pregnancy loss. Based on these findings, we recommend policy measures aimed at reducing pregnancy loss Sub-Saharan African countries.

## Introduction

Pregnancy loss refers to the end of a pregnancy before the fetus can survive outside the womb. It can occur spontaneously or be induced for medical or personal reasons. The main types of pregnancy loss include miscarriage, abortion, and stillbirth (1, 2). Pregnancy loss is a significant public health issue globally, particularly in Sub-Saharan Africa (SSA) (3–5). Worldwide, there are thought to be 73 million induced abortions performed annually. Approximately 45% of abortions are unsafe, and 97% of those occur in developing nations. In 2022, the global late adolescent birth rate for girls was estimated to be 1.5 per 1,000 women, with higher rates in SSA (6).

In Sub-Saharan Africa, the rate of unwanted pregnancies was 91 per 1,000 women, compared to 35 per 1,000 in Europe and North America for women aged 15–49 (7). Between 1990 and 1994 and 2015–2019, the proportion of unintended pregnancies resolved through abortion increased by 26% in Middle Africa, 44% in Eastern and Western Africa, and 72% in Southern Africa (8).

One of the major reasons for high fertility rates in most SSA countries is that a significant number of women in SSA do not use contraception (9). One of the main causes of maternal deaths and morbidities is unsafe abortion (10). It may result in issues with women's physical and mental health as well by way of financial and social hardships for them, their communities, and the impact on families and healthcare systems (11).

Numerous studies had revealed barriers to pregnancy loss, such as insufficient understanding of family planning techniques and their accessibility, poor quality and restricted family planning service availability, and exorbitant expenses associated with family planning techniques, services, travel, and time (12–16).

Furthermore, previous research has found that the most important predictors of lower odds of pregnancy loss were having knowledge of modern contraceptive methods, marriage, and women with higher education (7, 17, 18). Moreover, studies showed that women from lower-income backgrounds had faced challenges in accessing prenatal care or safe reproductive health services, leading to higher risks of (19).

Currently, there is a gap in the literature based on recent data from East African countries. Classical statistical methods Although these models can provide valuable insights, their reliance on predefined rules and assumptions limits them (19–21), which can be limited in capturing complex, nonlinear interactions between variables, lack of automated methods for model selection and optimization, lack of flexibility to efficiently handle high-dimensional data, and patterns.

In contrast, machine learning models have the potential to improve predictive accuracy by learning from larger, more diverse datasets and identifying hidden patterns that traditional models may overlook (22, 23). Additionally, Machine learning (ML) models, on the other hand, have gained attention for their ability to automatically learn from data, capture these complex patterns, and make more accurate predictions (24).

The ultimate goal of this study is to contribute to improved reproductive health outcomes in Sub-Saharan Africa by providing actionable insights into the predictors of pregnancy. Through the use of machine learning, we aim to create a data-driven tool that can help health professionals predict and address the needs of at-risk populations while also offering evidence to support more effective and equitable care. Therefore, by overcoming the above limitations’, this study aimed to predict pregnancy loss and identify its determinants among women of reproductive age in SSA, using a machine learning algorithm using the most recent demographic and health survey dataset.

## Methods and materials

### Study design and study period

**Figure 1:**
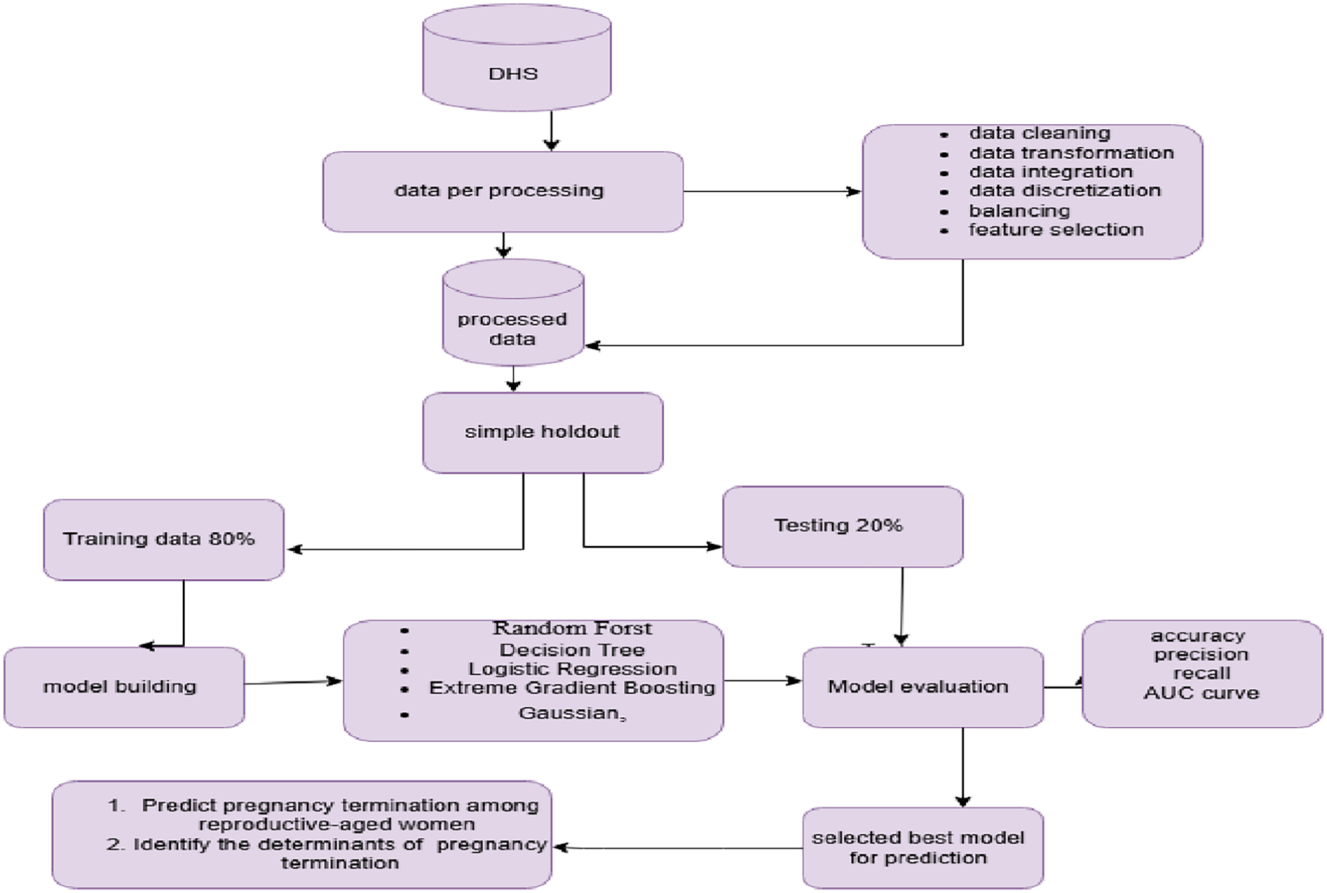
Flow diagram for data analysis and model building to predict pregnancy loss.

This study adopted a design science approach for further analysis of the DHS, which was conducted from 2012 to 2023. The design science approach focuses on creating and evaluating artifacts (such as models, methods, constructs, or systems) to solve complex, real-world problems. Unlike traditional research, which may focus solely on theory-building or hypothesis testing, the design science approach emphasizes the design and innovation of solutions to address specific issues or needs (25) show in Figure 1.

### Study setting

This study used the most recent dataset from the Demographic and Health Survey (DHS) for SSA. The dataset spans from 2012 to 2023, and the study covers 36 selected SSA. sub-Saharan Africa is regionally classified into West Africa, Southern Africa, East Africa, and Central Africa: East Africa (Burundi, Comoros, Ethiopia, Kenya, Madagascar, Malawi, Mozambique, Rwanda, Tanzania, Uganda, Zambia, and Zimbabwe), West Africa (Burkina Faso, Benin, Côte d'Ivoire, Ghana, Guinea, Liberia, Mali, Nigeria, Niger, Sierra Leone, Senegal, and Togo), Southern Africa (Lesotho, Namibia, Eswatini, and South Africa), and Central Africa (Angola, Democratic Republic of the Congo, Cameroon, Chad, Gabon, São Tomé and Príncipe, and Zambia).

### Population, and eligibility criteria

This study included reproductive-age (15–49 years) women who had experienced pregnancy loss in the selected enumeration areas at the time of DHS data collection.

### Data source

This study used the most recent DHS datasets obtained from the DHS Program website (http://www.dhsprogram.com) after permission was granted, following the submission of the study's justification and project title. The Women's Record (IR) dataset was used for this investigation. A two-stage probability sampling method, stratified by geographic region and urban/rural areas within each region, was applied to select study participants, ensuring the sample fully represents the target population of Sub-Saharan African countries. After data preparation, a weighted sample of 425,810 reproductive-age women was included in the study.

### Sample size and sampling procedure

This study used a weighted sample of 425,810 reproductive-age women. Due to the non-proportional distribution of the sample size across different regions, variations between urban and rural areas, and potential differences in response rates, sampling weights were applied to maintain representativeness. Participants were selected using a two-stage stratified cluster sampling procedure.

In the first stage, Enumeration Areas (EAs) were randomly selected based on their clusters. A stratified sample of census EAs from both urban and rural areas was chosen with complete household listings, using systematic probability sampling. This sampling was based on the sampling frame containing population and household information from the Population and Housing Census (PHC). In the second stage, households within the selected EAs were chosen using equal probability systematic sampling. In each selected household, reproductive-aged women were interviewed using an individual questionnaire (26).

### Study variables and measurements

#### Dependent variable

The dependent variable in this study was pregnancy loss among reproductive-aged women who experienced either miscarriage, abortion, or stillbirth that was dichotomized into two: “Yes” = 1 (if a woman encountered at least one of the three within 5 years preceding each survey, she is considered to have terminated pregnancy) and “No” = 0 if none of these events occurred.

#### Independent variables

In this study, we employed the fast recursive feature selection method to select the independent features.

### Operational definition

#### Pregnancy loss

Pregnancy loss is generally used to refer to the ending of a pregnancy, whether intentional or accidental. It encompasses a broad range of situations, including miscarriage, abortion, and stillbirth.

#### Unsafe abortion

It refers to the of a pregnancy performed under conditions that do not meet established medical standards of care, leading to an increased risk of complications such as infection, hemorrhage, and injury to the reproductive organs.

#### Stillbirth

It refers to the death of a fetus at or after 20 weeks of gestation, but before or during birth. This includes any fetal death occurring before or during the labor and delivery process.

Miscarriage, which is referred to as spontaneous abortion**,** is the early loss of a fetus before the 20th week of gestation.

#### Data analysis procedure

This study used data from the DHS of SSA to predict pregnancy loss and identify its factors among women of reproductive age. We utilized Python and several key libraries, including Pandas, scikit-learn, imbalance-learn (lmblearn), numPy, and matplotlib, for data preparation, model development, model construction, model evaluation, and model deployment. In conclusion, we developed a predictive model that forecasts pregnancy loss and identifies its determinants.

#### Data pre-processing

Data processing is a machine-learning technique that transforms raw data into an understandable format (27). In this study, we employed the major data preprocessing steps, which included dimensionality reduction, data transformation, data discretization, data integration, and data cleaning. The advantages of data preprocessing include improving model accuracy through tasks such as data cleaning, exploratory data analysis, normalization, dimensionality reduction, data transformation, and data integration, all of which can positively impact the model's performance (28).

#### Data cleaning

In this study, we employed data cleaning to address outlier values, imbalanced outcome variables, noise, and missing values. Raw data cannot be directly used in the model testing and training process, as it often contains missing values, which can lead to biased or inaccurate results (29). A large fraction of the 425,810 records had fewer than 643 missing data points for the critical features, comprising nearly 6.6% of the dataset. Our dataset contains missing values for both categorical and continuous variables. Features such as maternal age: 3.2%, birth order: 2.3%, and wealth index: 1.1% have missing values. We used mean imputation for continuous variables and mode imputation for categorical variables to address these missing values.

Outliers are data points that differ significantly from other observations in the dataset. In this study, we identified outliers using visualization techniques such as box plots and Z-scores. To remove outliers, we use methods for identifying outliers by using the Z-score. The threshold is a Z-score greater than 3 or less than −3. This indicates that the data point is more than 3 standard deviations away from the mean and is therefore an outlier.

#### Feature selection

Feature selection is the process of removing irrelevant or redundant features during the development of a predictive model (30). Feature selection methods were applied during data preprocessing to achieve more efficient data. Furthermore, this study includes 48 features, making feature selection essential. Excessive features are time-consuming and resource-intensive, so it is important to speed up model building and improve the model's performance (31). As a result, identifying the most important features associated with pregnancy loss is a fundamental step. In this study, we employed Recursive Feature Elimination (RFE) to identify the most relevant variables for predicting pregnancy loss. Since RFE infers the relevance of features by estimating their importance through the algorithm, it selects the most important features.

#### Data transformation

Data transformation involves converting the data into a format suitable for analysis; this may include changing the data's types, scaling, normalizing, and renaming (32). In this study, we used the one-hot encoding technique to convert string data into integers, ensuring a uniform data type for machine learning classifiers. Before building the model, we also scaled the dataset to standardize it, making it suitable for analysis and improving model training and evaluation.

#### Data discretization

Data discretization is the method of converting continuous data (numerical values) into discrete categories or intervals (33). In this study, we employ binning as a technique to enhance the interpretability and performance of classification algorithms by transforming continuous input into categorical input. Data discretization was used, which limits the impact of outliers, makes it easier to analyze, and reduces noise by transforming continuous variables into categorical features to make the data easier to understand and analyze. For example, reproductive mother's age is continuous; attributes were discretized into 15–24, 25–34, and 35–49 according to DHS guidelines (34, 35).

#### Data standardization and data integration

This study uses the “standard Scaler ()” library of “sklearn” to normalize the data in order to avoid scaling issues for distance-based learning approaches like logistic regression and gaussian. In this study, we used 36 sub-Saharan African countries DHS datasets, integrated them based on identification variables, sorted both data files by the identification variables, determined the base (primary) file, and finally merged them using Python software.

#### Class balancing

Before training the prediction model, an unbalanced dataset was balancing (resampled); this might be viewed as a data preparation step (36). Synthetic Minority Oversampling (SMOTE) was used to balance the training data in order to prevent machine learning models from being biased toward the majority class (37). We utilized SMOTE oversampling by creating synthetic examples (new observations) that resemble the minority class by interpolating between minority classes samples in the feature space rather than creating exact copies of existing examples. SMOTE has been shown to almost continually increase classification model performance for resampling imbalanced datasets (38).

#### Model selection

The predicted variable in this study was binary classification, since pregnancy loss was divided into two “yes” or “no.” For model building, four classifiers: fandom forests, Boost, Gaussian, and decision trees were used. The algorithms were chosen in accordance with previous research that used machine-learning methods to classify tasks (39, 40). Machine learning has been widely used to predict childbirth mode and assess potential maternal risks during pregnancy (24). The rationale behind the selected algorithm in this study is its ease of implementation, interpretability, training efficiency, reduction of over fitting, and speed in predicting unknown records (41, 42).

Our aim for this study is to apply ML for pregnancy loss among reproductive-aged women and to provide insight for the government and policymakers. Random forests, XGBoost, decision trees, and Gaussian were used to identify the predictors due to their favorable prediction performance in prior research (24, 43, 44).

#### Decision tree

We're using an entirely novel integrated supervised learning algorithm developed for this study to effectively handle enormous volumes of survey data. Because it is easy to use and predictable, the study's methodology combines theory and practice in a novel and innovative way. One of the most widely used methods for representing predictions is the decision tree (45). Because decision trees are easily interpretable and can efficiently deal with large, complicated datasets without imposing a complicated parametric structure, they can be quite powerful when used in ensemble algorithms and are resistant to outliers.

#### Random forest

Random forest is a machine learning algorithm that ensembles multiple decision trees to make predictions for classification and regression problems (46). The concept of multiple random tree generation is used in each split decision, along with a voting system, sample bagging, training bootstrapping, and randomly selected features. Random Forest overcomes these limitations of decision trees by using an ensemble of decision trees (46). Due to an ensemble of models, bootstrap aggregating, sometimes known as bagging, increases prediction accuracy and stability.

#### Gradient boosting

Extreme Gradient Boosting (XGBoost) is a decision-tree-based ensemble machine learning algorithm that uses a gradient boosting framework to improve the speed and efficiency of boosted tree algorithms (47). It can manage the issue of over fitting, but scalability on larger datasets is a concern due to its sensitivity to outliers.

#### Data splitting

In this study, we utilized a simple holdout method, with 80% (340,648 samples) for training and 20% (85,162 samples) for testing, to ensure robust model evaluation. The dataset was divided into training and testing sets, with the training set used to build and train the model and the testing set used to evaluate its performance on previously unseen data.

#### Model evaluation

The performance of the trained model is evaluated using the testing dataset. Then, the performance of the trained models was evaluated using the test set based on the criteria of accuracy score, AUC curve, precision (P), recall (R), and F-measure as follows: The confusion matrix is a matrix of N * N, where N is the number of predicted classes, and it displays the number of correct (48) and incorrect predictions made by the classification model relative to the target value. Subsequently, the test set was used to assess the trained models’ performance using the accuracy score, AUC curve, precision (P), recall (R), and F-measure criteria.

(1) Precision=(TP)/(TP+FP)

(2) Recall=(TP)/(TP+FN)

(3) F–Measure=(2*Precision*Recall)/(Precision+Recall)

(4) Accuracy=((TP+TN)/TP+TN+FP+FN))×100

## Results

### Socio demographic characteristics of the study participant

This study investigated a sample of 425,810 reproductive-aged women from 36 countries in Sub-Saharan Africa, all of which were part of a Demographic and Health Survey. The results showed that 7% of women had a pregnancy loss, while 93% had not. As shown in Table 1 women who had never married (0.6%) had a lower prevalence of pregnancy loss compared to women who were married, who had a higher pregnancy loss rate (6%). Among women who had experienced a pregnancy loss, approximately 5% (22, 49) of women who had used contraceptives experienced pregnancy loss, compared to 12% (5,885) of women who had not used contraceptives. Furthermore, women who were exposed to watching TV (53%) had a lower rate of pregnancy loss, whereas 34% of women who did not watch TV had a higher prevalence of pregnancy loss (10%).

**Table 1:** Socio demographic characteristics of the study participant, evidences from DHS (*N* = 425,810).

Variable	Frequency (%)	Pregnancy loss(PT)	
		YES	NO
Marital status			
Never married	116,903 (64%)	2,784 (0.6%)	114,119 (27%)
Married	273,548 (28%)	23,855 (6%)	249,693 (58%)
Widowed, divorced and separated	35,359 (8)	2,424 (0.5)	32,935 (8)
Contraceptive use			
Yes	342,402 (80%)	23,178 (5%)	319,224 (75%)
No	83,408 (20%)	5,885 (12%)	319,224 (18%)
Watching TV			
Yes	240,766 (56%)	14,827 (4%)	225,939 (53%)
No	185,044 (44%)	14,236 (10%)	170,808 (34%)
Parity			
Null parity	114,951 (27%)	4,194 (1%)	110,757 (26%)
Prim parity	59,203 (14%)	5,445 (2%)	53,758 (12%)
Multi parity	143,454 (34%)	11,945 (3%)	131,509 (31%)
Grand parity	108,202 (25)	7,479 (2%)	100,723 (23%)
Maternal age			
15–24 years	169,095 (40%)	8,239 (1%)	160,856 (39%)
25–34 years	133,148 (31%)	12,191 (3%)	120,957 (27%)
35–49 years	123,567 (29%)	8,633 (2%)	114,934 (27%)
Independence			
Living Alone	43,076 (10)	3,830 (1%)	39,246 (9%)
Living with jointly with husband	100,939 (24%)	8,863 (2%)	92,075 (21%)
Living with others	281,796(66%)	1,670(4%)	265,426(62%)

#### Class balancing

**Figure 2:**
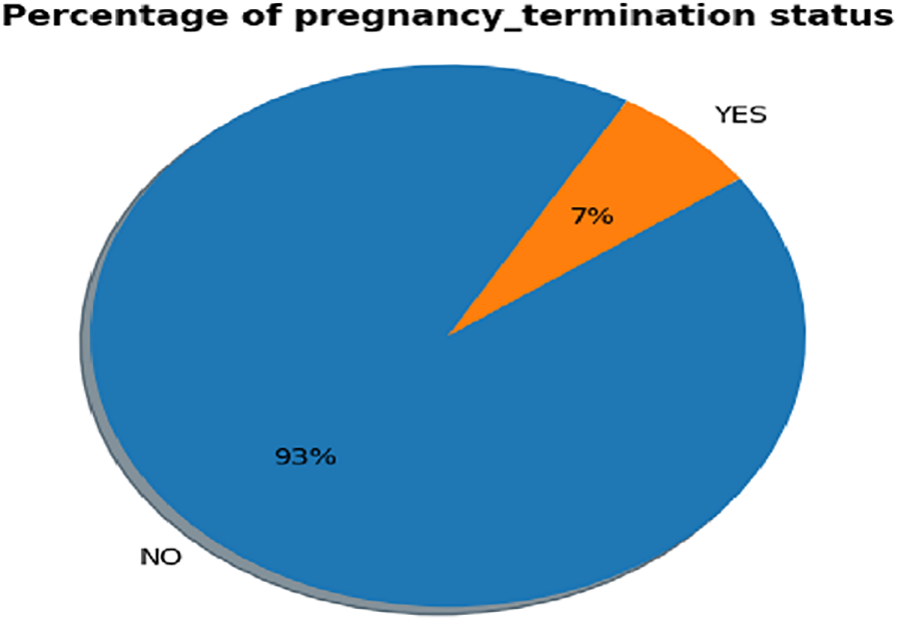
Prevalence of pregnancy loss status among reprodactive-aged women.

In this study, 29,806 (7%) women had a pregnancy loss, while 396,004 (93%) did not, as shown in Figure 2. To prevent machine learning models from being biased toward the majority class, the training data was balanced using the SMOTE. The synthetic minority oversampling technique was applied to overcome sampling bias by generating new, synthetic observations that approximate the minority class. This is done by interpolating between minority-class samples in the feature space rather than simply replicating existing examples. As a result, the distribution of the classes was balanced, creating an equal and symmetric distribution in each category, which helps build more reliable predictive models. We obtained a balanced sample of women who had a pregnancy loss with counts 396,004 and not with count 396,004.

#### Importance feature selection

**Figure 3:**
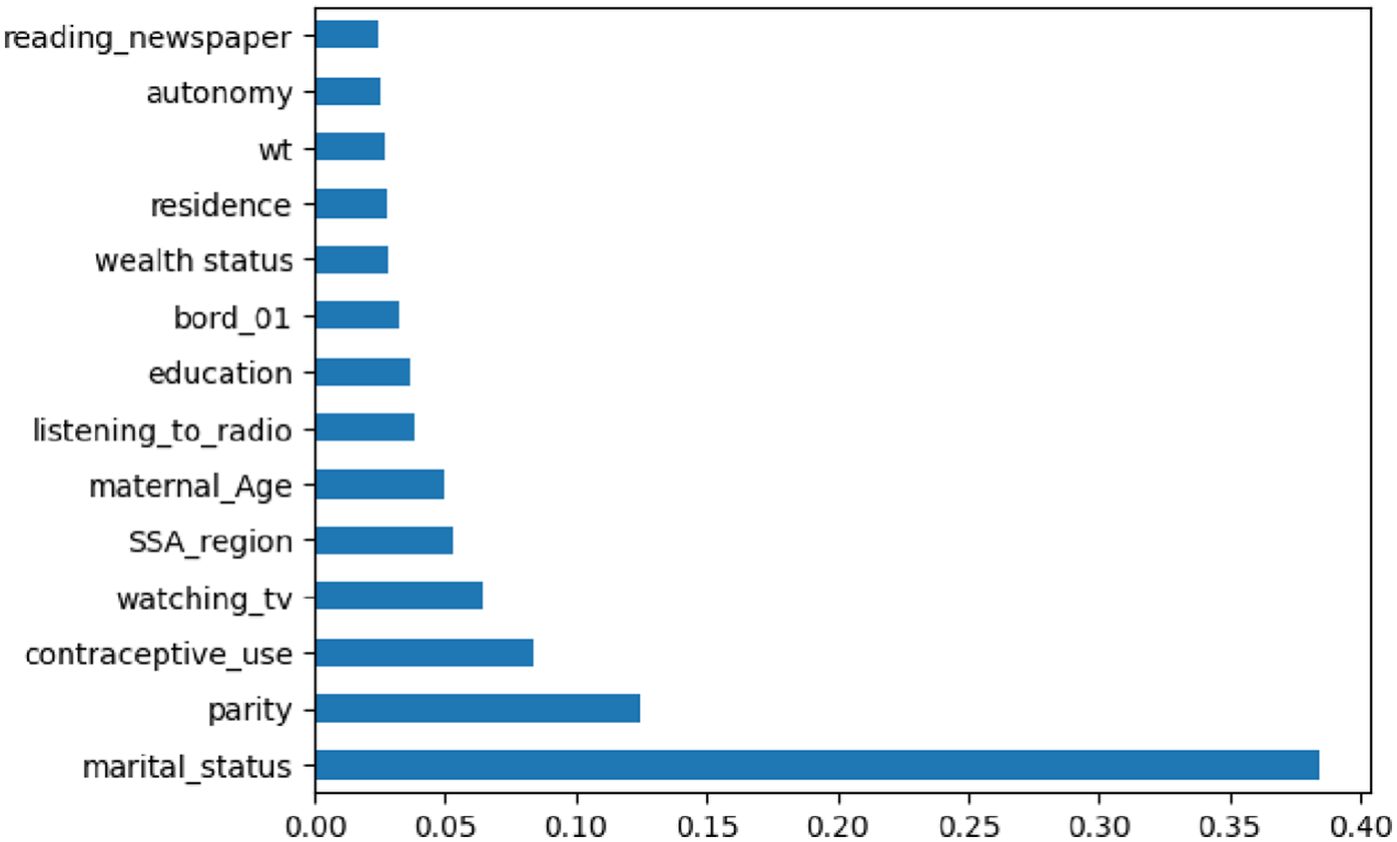
Important features selected by recursive feature elimination (RFE).

Feature selection and variable importance ranking are techniques used to identify a subset of relevant features by removing irrelevant or redundant ones. The importance of feature selection lies in its ability to reduce the cost of learning by decreasing the number of features, improving model performance, and minimizing storage and computation time. The dataset contains 48 features. To select the most important ones, we employed RFE. This approach provides flexibility in controlling the number of features retained and can effectively handle correlated features. As shown in Figure 3, the selected features included marital status, parity, contraceptive use, wealth status, residence, birth order, maternal age, reading newspapers, listening to the radio, country, watching TV, weight, and independence. These determinants were used for model building.

#### Comparisons of selected machine learning model

**Figure 4:**
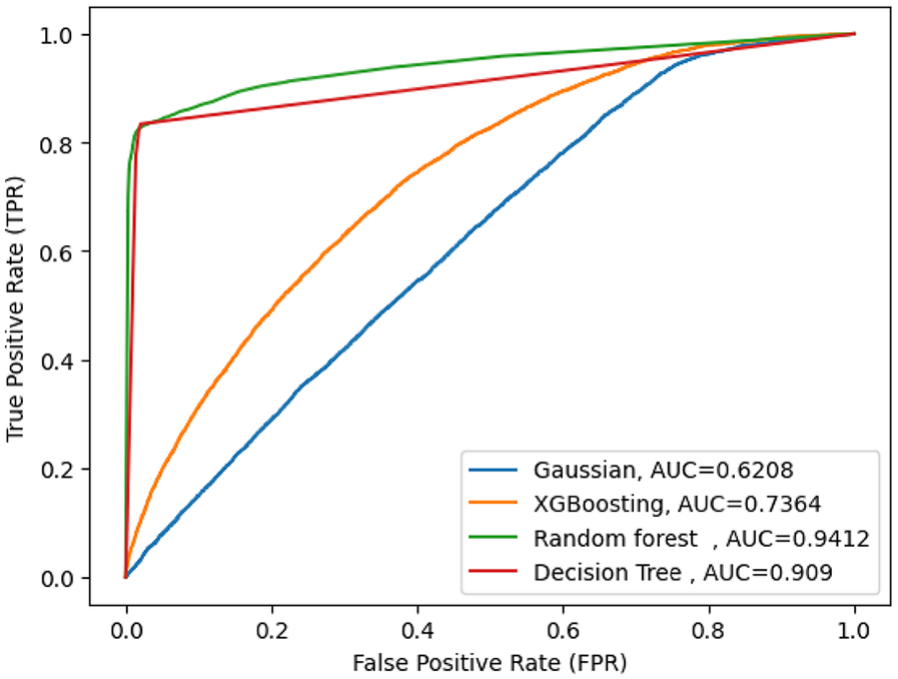
AUC curve score of machine learning model.

In order to identify determinants and predict pregnancy loss among reproductive-aged women, the study used supervised machine learning techniques such as logistic regression, decision tree (DT), XGB (Extreme Gradient Boosting), and Gaussian Naive Bayes for analysis. The study compared these machine learning methods using the same testing parameters to build predictive models. The evaluation metrics included accuracy, Area under the Curve (AUC), precision, recall, and F-measure. Since Random Forest (RF) performed the best overall, it was chosen as the top model. The results are displayed in Figure 4 and Table 2. Random Forest achieved an accuracy of 98%, precision of 98%, F-measure of 83%, ROC curve of 94%, and recall of 77%. Additionally, Random Forest had a high true positive rate (99.3%), a low false positive rate (2%), a true negative rate of 94.6%, and an extremely low false negative rate (0.006%). The AUC for Random Forest was 94%. The Decision Tree model had an accuracy of 97.19%, precision of 82%, recall of 78%, F-measure of 80%, and an ROC curve of 90%. Among the proposed models, the Gaussian Naive Bayes classifier performed the least well, with an accuracy of 92.9%, precision of 12.3%, recall of 50%, F-measure of 10%, and an AUC curve of 62%.

**Table 2:** Accuracy, precision, recall and F-measure for the machine learning algorithms.

ML model	Accuracy	Precision	Recall	F-measure	AUC
Random forest(RF)	98%	98%	77%	83%	94%
DecisionTree (DT)	97.19%	82%	78%	80%	90%
XGB(Extreme Gradient Boosting)	93.17%	92%	18%	3.7%	73%
Gaussian	92.92%	12.3%	0.5%	10%	62%

#### Model explanation using SHAP values

Interpreting the results of machine learning algorithms can be significantly more challenging than traditional statistical analysis methods. It is often difficult to understand how predictions are made. However, techniques like SHAP (Shapley Additive Explanations), proposed by Lundberg and Lee, provide a unified framework for interpreting the outputs of a wide range of machine learning models. SHAP values are used to gain insights into the contributions of individual features to the model's predictions, helping to explain how each feature influences the final decision (20).

In this study, we employed the Random Forest classifier in combination with the model-agnostic SHAP technique to identify the most significant predictors of pregnancy loss. By evaluating the mean absolute SHAP values across the dataset, we were able to identify the primary predictors for women who had experienced a miscarriage, abortion, or stillbirth.

#### The positive contributions (in red) features for women had none of a miscarriage, abortion or stillbirth

**Figure 5:**
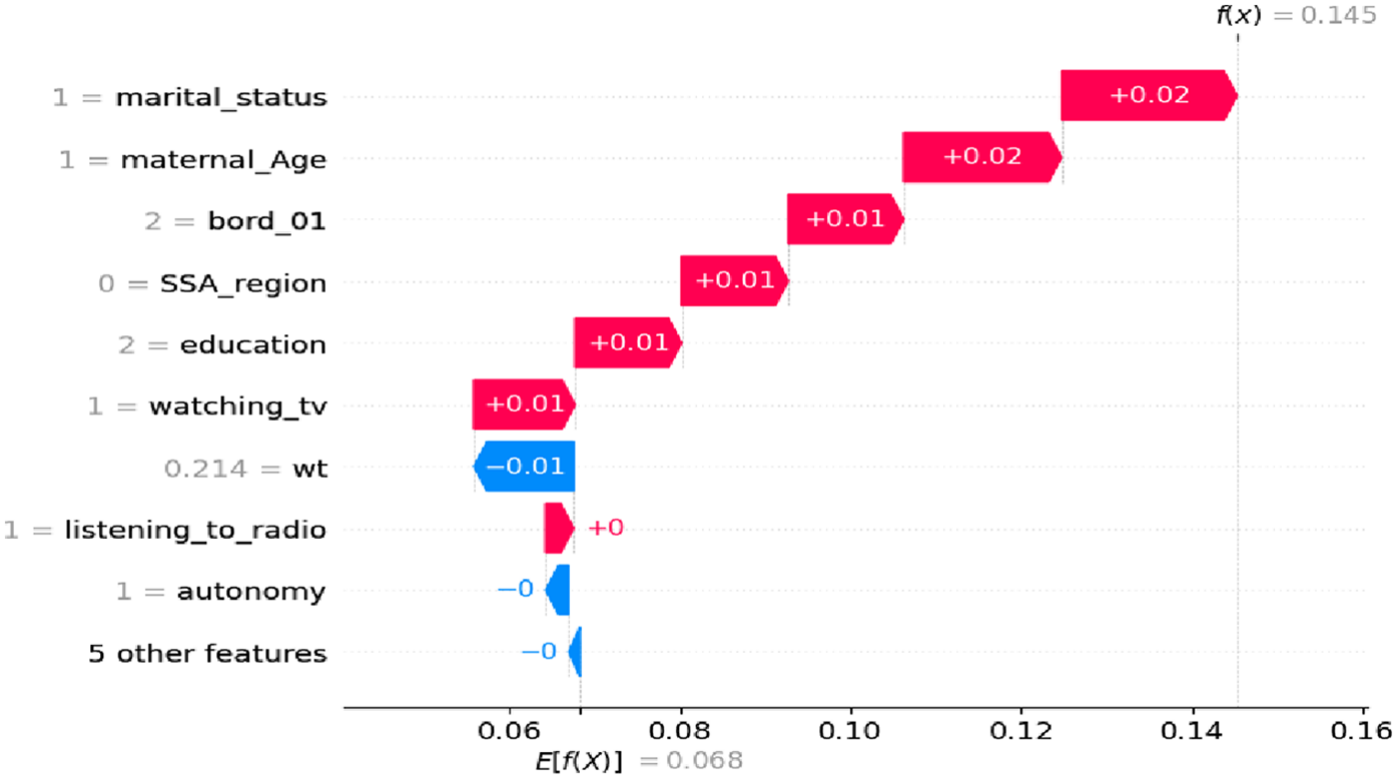
Waterfall plot displaying prediction of the 5th index observation high value.

As shown in Figure 5, where the SHAP values are positive, the features contribute to women who have not experienced a miscarriage, abortion, or stillbirth. This is represented by the red line, indicating the category coded as ‘1’ or a high value. These features tend to increase the predicted likelihood of not terminating a pregnancy. Examples of these features include being a married woman, being in the 25–34 maternal age range, having a second birth order, having secondary or higher education, watching TV, and using modern contraception methods. In total, five features have a positive impact on reproductive-aged women who have not experienced miscarriage, abortion, or stillbirth.

#### The negative contributions (in blue) features for women had experienced a miscarriage, abortion or stillbirth

**Figure 6:**
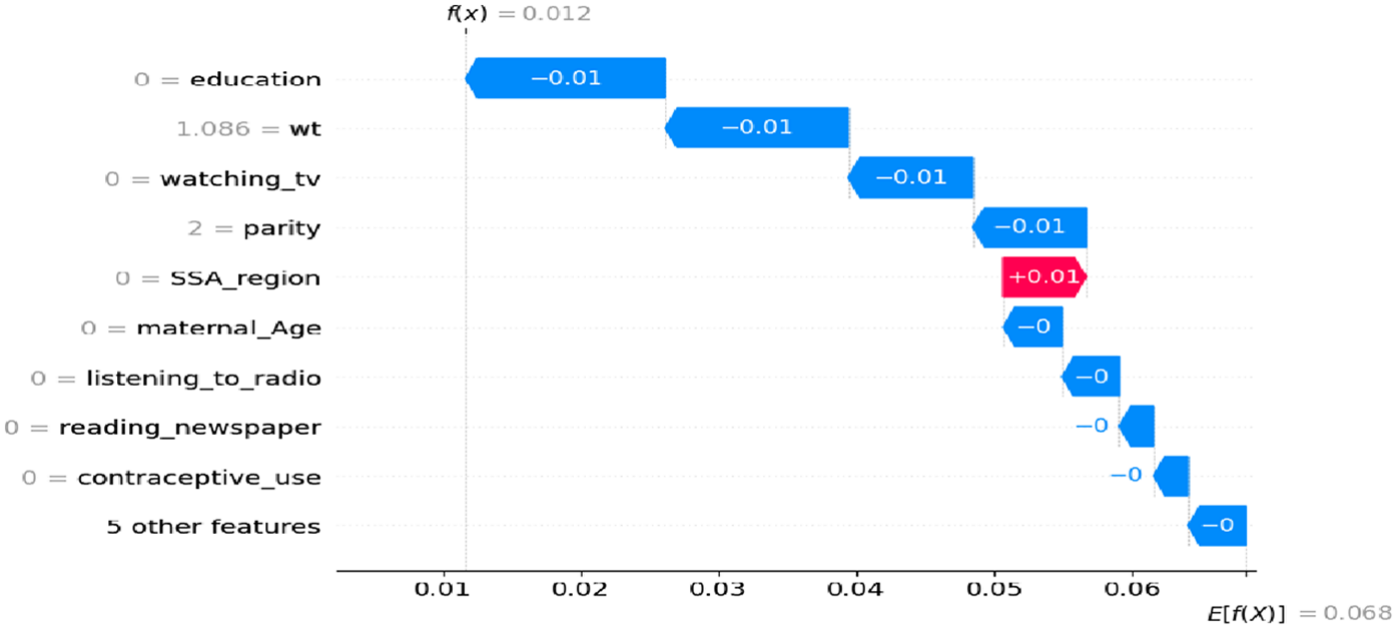
Waterfall plot displaying prediction of the zero index observation for low values.

As shown in Figure 6, where the SHAP values are negative, this is represented by the blue line, indicating the category coded as ‘0’ (no) or a low value. Increasing the value of these features tends to increase the likelihood of pregnancy loss (such as a miscarriage, abortion, or stillbirth). For example, women who are uneducated, have no experience with watching TV, have given birth to two children, are in the 15–24 maternal age range, do not use modern contraception methods, and have no media exposure appear to have a negative impact on pregnancy outcomes.

## Discussion

This study used a classification machine learning method to compare, identify, and recognize specific risk factors related to pregnancy loss among reproductive-aged women in SSA, which could be targeted for intervention. When compared to other machine learning classifiers, such as Decision Tree (DT), Extreme Gradient Boosting (XGB), and Naïve Bayes Gaussian, the Random Forest (RF) model demonstrated the best predictive power for identifying risk factors for pregnancy loss. It achieved an accuracy of 98.0%, recall of 77.0%, precision of 98.0%, F1 score of 98.0%, and AUC of 94%. The feature importance analysis, performed using RFE, identified the following as critical predictors of pregnancy loss in sub-Saharan Africa countries: marital status, parity, contraceptive use, wealth status, place of residence, birth order, maternal age, reading newspapers, listening to the radio, watching TV, weight, and independence.

Some of these variables had already been proven to be predictors of pregnancy loss in previously published studies. The first important feature of SSA predictors of pregnancy loss was marital status. We found that never married women were more likely to report having a pregnancy loss compared with those who were married. This could be due to a tendency toward a delay in women's age at marriage, which has been suggested to result in increased sexual activity among never-married women and raise their risk of unintended pregnancy. These women may also obtain a pregnancy because they feel that having a child would interfere with future opportunities. Yet, fear of school dropout was mentioned as a primary reason for demanding an induced abortion service among women of reproductive age in SSA (15, 20). Younger women and those who are unmarried were found to have higher probabilities of pregnancy loss (50).

With regard to parity, multiparous women were less likely to experience pregnancy loss compared to primiparous women. This may be because women without children are more likely to be teenagers, and the likelihood of unwanted pregnancies, which often result in abortion, is higher among young women, due in part to an unmet demand for family planning (17, 51). Limited access to contraception and family planning services was a major factor influencing the decision to terminate a pregnancy. Areas with high unmet need for family planning, especially in rural regions, saw higher rates of pregnancy loss, as women may not have had the opportunity to prevent unintended pregnancies in the first place (52).

Women who had media exposure were less likely to experience pregnancy loss compared to those without. This could be due to the fact that the media plays a vital role in broadcasting information about how and where to end a pregnancy. Additionally, women exposed to media may be better informed about abortion-related principles and are potentially less likely to face societal stigma (19, 53).

Women with lower incomes had higher odds of having an induced abortion compared to those with higher incomes (20, 54, 55), thus, the low contraceptive utilization among women with lower income may account for the higher odds of induced abortion in this study. In turn, studies have demonstrated that women who use contraceptives are less likely to have an induced abortion compared to those who do not (17, 20, 56).

This study showed that pregnant women who had secondary and above educational levels were more likely to undergo pregnancy loss as compared with those with no education. A similar study conducted in Ethiopia showed that the likelihood of pregnancy loss in uneducated women was 1.5 times lower than in women who attended elementary school, 1.5 times lower than in women who attended secondary school, and 1.8 times lower than in women who attended higher education (56). The reason for this is that women who have knowledge about the fertile period are 35% less likely to have an unsafe abortion compared to those with no knowledge of the fertile period. This is because educated women are more likely to use modern contraceptive methods to prevent unwanted pregnancies (57).

The goal of this study was to use supervised machine learning algorithms to predict pregnancy loss and identify the key determinants among reproductive-aged women in Sub-Saharan Africa. By leveraging the potential of machine learning models, we aimed to provide insights that can assist in improving maternal health policies, healthcare services, and intervention strategies in this SSA.

### Strength and limitations

This study attempted to forecast pregnancy loss and more accurately evaluate the key predictors. Also, this study made use of the recent DHS data set of sub-Saharan Africa countries, which contains almost every demographic risk group that is vulnerable. However, this study has certain limitations because the DHS data collection is self-reported, which may have introduced some information biases.

## Conclusion and recommendation

Machine learning can play a significant role in predicting pregnancy loss and understanding the underlying factors among reproductive-aged women in Sub-Saharan Africa. By leveraging data on demographics, health, and socio-economic status, predictive models can help policymakers and healthcare workers to better anticipate and address pregnancy-related complications, improve maternal health outcomes, and allocate resources more effectively. The finding is very important to identifying factors such as lack of prenatal care or socio-economic barriers that can help design more effective prevention programs. However, future research could explore the integration of more granular and real-time data sources, such as electronic health records and mobile health applications, to enhance prediction accuracy. Additionally, expanding the scope of machine learning models to account for cultural, legal, and regional variations in pregnancy-related outcomes could further improve the applicability and generalizability of these models across diverse populations in SSA.

## Data Availability

The datasets presented in this study can be found in online repositories. The names of the repository/repositories and accession number(s) can be found below: http://www.dhsprogram.com.
